# Ionic Treatment of Dental Sepsis

**Published:** 1914-01-10

**Authors:** 


### IONIC TREATMENT FOR DENTAL SEPSIS.

### The Prevalence and Prevention of the Disease.

In the Section of Stomatology at the last International Congress of Medicine much attention was paid—and quite rightly—to that exceedingly prevalent condition of suppuration in the gums and tooth sockets which is known technically as pyorrhœa alveolaris. The increasing care which physicians have been paying of late years to the mouths of their patients has made it fairly clear that this condition of pyorrhœa alveolaris is responsible for an enormous amount of ill-health, suffering, and even death, amongst all classes. Gastric and other ulcers, blood-poisoning of various kinds, and scores of other serious maladies have been traced in a certain percentage of cases to the chronic production and swallowing of matter from neglected teeth or roots or gums.

At the sessions of the Congress, discussions on this fearfully common, but easily preventable disease, were carried on. Rightly, the speakers dealt especially with prevention, which is a good deal more important than cure. Nevertheless, cure is important too, because the disease is so widely spread amongst all classes, but especially amongst those who cannot afford to pay for good advice as to how the condition can be ameliorated and its attendant evils mitigated. Many doctors and dentists set out to cure pyorrhœa by the drastic process of removing all the teeth and stumps round which suppuration is taking place. But as it frequently happens that the teeth themselves are quite good, it is plain that any more conservative method which is equally efficacious will be preferable. In the *Midland Medical Journal*, Mr. G. F. C. Matthews contributes a brief article on this topic, in which he gives his experiences of the ionic treatment which has been advocated especially by Sturridge and by Webster.

Mr. Matthews begins by noting the alarming increase of the disease in recent times. We are not so sure that this increase is actual; it may be that more notice is taken of it, and so it is more frequently looked for and discovered. He also states that it is more prevalent in some districts than in others, and that it is mostly a disease of middle life. These observations would tend to support the view that sheer neglect and dental uncleanliness are not the only factors at work in the production of pyorrhœa, though Mr. Matthews does believe that disregard of oral hygiene is the main and the commonest cause. The disease sometimes comes on after acute general infections, such as influenza; and the present writer has seen it follow septicæmia in a medical man who fully realised the benefits of oral antisepsis.

The technique of the treatment which Mr. Matthews recommends is simple. At the first visit the teeth are cleansed and all visible tartar removed. The teeth are polished, and the patient instructed as to the correct usage of tooth brush, mouth wash, etc. Every care is taken to avoid injury to the gum edges.

At the next visit, the pockets in the gums are freed from all food débris and similar accumulations, and packed with cotton-wool saturated in a 2-per-cent. solution of zinc sulphate. The zinc electrode of a battery, wrapped in cotton saturated in the same solution of zinc sulphate, is passed into the packing. It is then connected with the positive pole of a battery and a current of half to two milliamperes is allowed to flow for a quarter of an hour. By this means deep sterilisation of the recesses of the pus-producing cavities is attained. On removing the packing, the pocket presents a pearly, glistening appearance if the dosage has been properly administered. Any further particles of tartar are then easily detected and removed.

At the next visit (on the next day, it must be understood) the gum should show marked improvement; only in extreme cases will it be possible to express any pus. Massage should be given by the dental surgeon, and the patient instructed to use a rubber brush. Two teeth may be ionised at a single sitting, massage being at the same time employed for those treated at any previous visit. Splinting and ligaturing of all loose teeth is essential before beginning treatment; in fact, it is only when the teeth are firm that the chance of cure without removing the teeth is good. It is of great importance that scaling shall be thorough, that all instruments used shall be properly sterilised, and that great patience be expended. The co-operation of the patient is also an absolute necessity, for only by the utmost cleanliness and attention to the dental toilet can re-infection and relapse be avoided. Massage, too, must be continued by the patient for a permanently satisfactory result.

If further experience confirms the value which Mr. Matthews attributes to this form of ionic medication, a considerable step forward will have been taken. Unfortunately, the classes most in need of its benefits can afford neither the money nor the time necessary for such thorough and elaborate methods. For the labouring classes prevention is, however, as feasible as it is for those in more affluent circumstances; and it is on this that reliance must be placed for the production of a higher standard of dental healthiness, and consequently of general health too. Prevention is, of course, mainly a matter of instruction and education; and it is only the young who are teachable. To convince the average adult labourer, or his women-folk, of the utility of tooth brushes and general attention to the mouth is almost a hopeless task. Even if flat disbelief is not actually expressed, it will at least exist to such an extent as to prevent any efficient measures of dental hygiene. But there is some hope of impressing the truth on the young during the school age; and probably there will be less pyorrhœa in thirty years' time than there is to-day.

